# Identification of RNA-Binding Proteins Regulating the Alternative Splicing of *TREM2* Exon 4

**DOI:** 10.3390/genes17101188

**Published:** 2026-09-25

**Authors:** Hina Yamada, Motoaki Yanaizu, Naoki Iwai, Masahide Kato, Hayato Suzuki, Mizuki Wakai, Makoto Araki, Yoshihiro Kino

**Affiliations:** 1 Department of RNA Pathobiology and Therapeutics, Meiji Pharmaceutical University, 2-522-1, Noshio, Kiyose-shi, Tokyo 204-8588, Japanm-yana@my-pharm.ac.jp (M.Y.);; 2 Department of Biochemistry, Meiji Pharmaceutical University, 2-522-1, Noshio, Kiyose-shi, Tokyo 204-8588, Japan

**Keywords:** TREM2, Alzheimer’s disease, alternative splicing, CELF proteins, sTREM2

## Abstract

Background/Objectives: Alzheimer’s disease (AD) is a neurodegenerative disorder characterized by progressive cognitive decline. Most AD cases are sporadic, with both environmental and genetic factors contributing to disease onset and progression. *TREM2*, an AD susceptibility gene, encodes a receptor protein expressed on the plasma membrane of microglia and macrophages. In addition to membrane-bound TREM2, soluble TREM2 (sTREM2) is also present in the brain. sTREM2 can be generated through two distinct mechanisms: proteolytic shedding of membrane-bound TREM2 and alternative splicing of *TREM2* exon 4. Because the mechanisms regulating the latter pathway remain unclear, we investigated the molecular mechanisms underlying *TREM2* exon 4 alternative splicing, with a particular focus on RNA-binding proteins (RBPs). Methods: We designed guide RNAs (gRNAs) targeting sequences within exon 4. The gRNAs and an exon 4 splicing reporter were transfected into HEK293-based cells with inducible dCasRx expression to identify regions containing cis-regulatory elements involved in exon 4 splicing. RBPs interacting with the candidate region were subsequently identified by RNA pulldown followed by LC–MS/MS analysis. Results: We identified regions within exon 4 containing putative cis-regulatory elements involved in its alternative splicing and found that CELF1, CELF2, and DDX41 interact with RNA containing one of these regions. Knockdown experiments indicated that these RBPs differentially regulate exon 4 splicing. Conclusions: Our findings suggest that *TREM2* exon 4 alternative splicing is regulated by multiple cis-regulatory elements and RBPs, including CELF1, CELF2, and DDX41. Together with our previous identification of CELF1 and CELF2 as regulators of *TREM2* exon 3 splicing, these findings suggest that CELF1 and CELF2 play complex roles in the regulation of *TREM2* alternative splicing.

## 1. Introduction

Alzheimer’s disease (AD) is a neurodegenerative disorder characterized by progressive cognitive decline and pathological hallmarks including the formation of senile plaques resulting from the aggregation and deposition of amyloid-β (Aβ) in the brain and neurofibrillary tangles composed of hyperphosphorylated tau protein [1]. In recent years, the role of microglia in AD has attracted increasing attention. Microglia are resident immune cells of the central nervous system and play important roles in maintaining brain homeostasis. In response to receptor–ligand interactions, microglia phagocytose Aβ [2]. They regulate neuroinflammation by releasing various cytokines and inflammatory mediators [3,4,5]. Furthermore, microglia have been reported to alter their gene expression profiles during the progression of AD pathology and transition into distinct states, including disease-associated microglia (DAM). DAM are localized around Aβ plaques and exhibit increased expression of genes associated with phagocytosis and lipid metabolism compared with homeostatic microglia [6].

Triggering receptor expressed on myeloid cells 2 (TREM2) is a transmembrane receptor protein expressed on the cell surface and is predominantly expressed by microglia in the brain, with particularly high expression in DAM [6,7]. In addition to microglia, TREM2 is also expressed in myeloid cells, including macrophages, dendritic cells, and osteoclasts [8,9,10]. TREM2 signaling is mediated through its interaction with the adaptor protein TYRO protein tyrosine kinase-binding protein (TYROBP) [9]. Upon binding of ligands, such as apolipoprotein E (APOE) [11,12], Aβ [13], and phospholipids [14], to the extracellular IgV domain of TREM2, tyrosine residues within the Immunoreceptor Tyrosine-based Activation Motif of TYROBP are phosphorylated, leading to the activation of downstream signaling pathways [15]. These downstream signaling pathways regulate various microglial functions, including Aβ degradation, depolarization, and activation [11,16]. The *TREM2* rs75932628-T variant has been identified as a risk variant for AD and results in the R47H substitution within the IgV domain [17,18].

TREM2 also exists in a soluble form, known as soluble TREM2 (sTREM2), which contains the ligand-binding IgV domain but lacks the transmembrane and cytoplasmic regions. In patients with mild cognitive impairment, the level of sTREM2 in the cerebrospinal fluid (CSF) is increased, and higher sTREM2 levels tend to be associated with a slower rate of cognitive decline [19,20]. Therefore, sTREM2 has attracted attention as a potential biomarker of AD [21,22,23]. Moreover, in a 5×FAD mouse model, sTREM2 has been reported to regulate microglial activity, attenuate Aβ pathology, and improve cognitive and synaptic functions [24,25]. Furthermore, higher CSF sTREM2 levels have been associated with reduced APOE4-related cognitive decline and a lower risk of AD-associated neurodegeneration, suggesting that sTREM2 may exert protective effects against AD pathology [20].

There are two major pathways for the production of sTREM2: proteolytic shedding of the extracellular domain of TREM2 and alternative splicing of *TREM2* mRNA. Proteolytic shedding is primarily mediated by ADAM10 and ADAM17, members of the A Disintegrin And Metalloprotease family, which cleave TREM2 at the H157-S158 site [26,27]. The other pathway involves alternative splicing of *TREM2* mRNA, which generates several splice variants. Among these transcripts, the exon 4 (ex 4)-lacking transcript encodes a TREM2 isoform that lacks the transmembrane domain and is therefore secreted as sTREM2. This transcript (ENST00000338469) has been detected in the human brain and accounts for approximately 20–25% of total *TREM2* mRNA [28,29].

RNA-binding proteins (RBPs) play important roles in regulating alternative splicing and thereby contribute to transcript and protein diversity. In our previous studies, we demonstrated that skipping of *TREM2* exon 3 (ex 3) generates a premature termination codon in ex 4, leading to *TREM2* mRNA degradation through nonsense-mediated mRNA decay (NMD) [30]. Although ex 3 skipping occurs in wild-type (WT) *TREM2*, the resulting transcripts are barely detectable because of their rapid degradation by NMD [30]. We further demonstrated that CELF1 and CELF2, two RBPs involved in alternative splicing regulation, regulate *TREM2* ex 3 splicing [31]. Both CELF1 and CELF2 promote ex 3 skipping; however, CELF2 exerts a stronger effect, leading to reduced TREM2 protein levels through enhanced ex 3 skipping. It is also notable that a genomic region containing *CELF1* has been identified as an AD risk locus and that reduced *CELF1* mRNA expression has been reported in patients with AD [32]. A genetic association between CELF2 and AD has also been suggested [33,34]. In addition, CELF2 has been reported to regulate tau splicing [35,36].

Although the functional importance of sTREM2 has been demonstrated, the mechanisms underlying its production through alternative splicing of ex 4 remain unclear. Therefore, this study aimed to identify cis-regulatory elements and RBPs involved in *TREM2* ex 4 alternative splicing. First, we investigated which region of *TREM2* ex 4 is involved in splicing regulation using dCasRx and gRNAs, which can sterically interfere with RNA–RBP interactions in a sequence-specific manner. After identifying a candidate region, we generated a series of deletion mutants to further narrow down the sequence containing the putative cis-regulatory element. We then performed LC-MS/MS analysis to identify RBPs that bind to this region. This analysis revealed CELF1, CELF2, and DDX41 as candidate RBPs.

## 2. Materials and Methods

### 2.1. Plasmid Construction

All plasmids generated in this study were verified by Sanger sequencing (Fasmac, Kanagawa, Japan). The primers used in this study are listed in Supplementary Table S1.

### 2.2. gRNA Construction

CasRx gRNA cloning backbone was a gift from Patrick Hsu (Addgene plasmid # 109053; http://n2t.net/addgene:109053 (accessed on 2 December 2020); RRID: Addgene_109053) [37]. Twelve gRNAs were generated as described in our previous study [38]. The gRNA-specific forward and reverse primers were annealed in Annealing Buffer (10 mM Tris–HCl, pH 8.0, 50 mM NaCl and 1 mM EDTA) diluted with nuclease-free water by incubation at 95 °C for 5 min. The resulting double-stranded oligonucleotides were ligated into the pXR003 vector digested with BbsI [37].

### 2.3. TREM2 ex3-5 Minigene and Deletion Mutants

*TREM2* ex3-5 minigene fragment was amplified from full-length (fl)-*TREM2* minigene as a template using KOD plus neo (TOYOBO, Tokyo, Japan) with BamHI-TREM2-ex3-Fw and SalI-TREM2-ex5-Rv primers. The amplified fragment was inserted into the BamHI and SalI sites of the pEGFP-C1 vector (Clontech, San Jose, CA, USA). A series of deletion mutants of the *TREM2* ex3-5-minigene was generated by PCR-based mutagenesis. DNA fragments corresponding to the upstream and downstream regions flanking the deleted region were amplified separately using specific primer sets. The upstream and downstream fragments were then fused by overlap extension PCR using TREM2-int3-Fw and SalI-TREM2-ex5-Rv primers. The resulting PCR fragment was inserted into the PmeI/PmaCI sites of the *TREM2* ex3-5 minigene.

### 2.4. fl-TREM2 Minigene and Deletion Mutants

The fl-*TREM2* minigene was constructed as previously described [30]. The fl-*TREM2* minigene deletion mutant (fl-*TREM2*-ex4-d2 minigene) was generated using similar methods described above. The resulting PCR fragment was inserted into the EcoRV/NotI sites of the fl-*TREM2* minigene.

### 2.5. EGFP-Tagged Expression Plasmids

EGFP-CELF1 and EGFP-CELF2 expression constructs were generated as previously described [31]. EGFP-DDX41 was amplified from a HEK293 cell cDNA library by PCR using BglII-DDX41-Fw and SalI-DDX41-Rv. The PCR product was digested with BglII/SalI and inserted into the BglII/SalI sites of pEGFP-C1.

### 2.6. Cell Culture and Cell Line Establishment

HEK293 (RCB1637) and THP-1 (RCB1189) cell lines were obtained from the RIKEN BioResource Research Center (Kyoto, Japan). The HMC3 cell line (EP-CL-0620) was obtained from Elabscience (Houston, TX, USA). HEK293 cells stably expressing the fl-*TREM2* minigene (fl-TREM2 cell line) and HEK293-dCasRx cells were established in our previous study [30,38]. HEK293 cells were cultured in Dulbecco’s modified Eagle’s medium (DMEM; Wako, Osaka, Japan) supplemented with 10% fetal bovine serum (FBS; Nichirei Bioscience, Tokyo, Japan) and 1% penicillin–streptomycin (Wako). THP-1 cells were cultured in RPMI 1640 medium containing GlutaMAX (Invitrogen, Waltham, MA, USA) supplemented with 10% FBS and 1% penicillin/streptomycin. The human microglial cell line HMC3 was cultured in Eagle’s Minimum Essential Medium (EMEM; Wako) supplemented with 10% FBS and 1% penicillin–streptomycin. All cells were maintained at 37 °C in a humidified atmosphere containing 5% CO_2_. To inhibit nonsense-mediated mRNA decay, cells were treated with 50 μg/mL cycloheximide (CHX; Wako) for 6 h at 37 °C.

### 2.7. Reverse Transcription-PCR (RT-PCR)

Cell-based splicing assays were performed as previously described [30]. Typically, 0.5 μg of plasmid was transfected into cells seeded in 12-well plates using Lipofectamine 2000 (Thermo Fisher Scientific, Waltham, MA, USA), and cells were harvested 48 h post-transfection. For HEK293-dCasRx cells, a total of 0.02 μg of minigene and 0.48 μg of each gRNA were co-transfected and harvested 72 h post-transfection. For fluorescence-activated cell sorting (FACS), fl-TREM2 cells were seeded in 6-well plates and transfected with 1.0 μg of EGFP-fused expression plasmid using Lipofectamine 2000 or PEIMAX (Polysciences, Warrington, PA, USA). Cells were harvested 48 h after transfection, and two wells of a 6-well plate were pooled for each sample. Harvested cells were subjected to FACS using MA900 (SONY, Tokyo, Japan). For knockdown experiments, fl-TREM2 cells and HMC3 cells were seeded in 12-well plates and transfected with 30 pmol of siRNA using Lipofectamine RNAiMAX (Thermo Fisher Scientific). Cells were harvested 72 h after transfection. siLuciferase (siluc; Bioneer, Daejeon, Republic of Korea) was used as a negative control. The siRNA sequences used in this study are listed in Supplementary Table S2.

Total RNA was extracted using the RNA Basic Kit (NIPPON Genetics, Tokyo, Japan) with DNase treatment (TaKaRa Bio, Shiga, Japan). RNA was reverse-transcribed using ReverTra Ace-α- (TOYOBO) with oligo(dT)20 primer and random primers. Equal amounts of total RNA were used for reverse transcription across all samples. RT-PCR was performed using Blend-Taq -plus- (TOYOBO) or Ex-Taq (TaKaRa Bio) with the primer sets listed in Supplementary Table S1. PCR amplicons were resolved by electrophoresis using either Agarose 1200 Standard Type (pH Japan, Hiroshima, Japan) or UltraPure Low Melting Point Agarose (Thermo Fisher Scientific). Gels were stained with ethidium bromide (NIPPON GENE, Tokyo, Japan). Images were captured using a Luminograph III (ATTO, Tokyo, Japan) and quantified using CSAnalyzer (ATTO). Original uncropped gel images are provided in Supplementary Figure S3.

### 2.8. Quantitative PCR (qPCR)

qPCR was performed as previously described [38]. Primer sequences used in this study are listed in Supplementary Table S1. β-Actin (*ACTB*) was used as the endogenous control, and relative gene expression levels were quantified using the standard curve method.

### 2.9. LC-MS/MS Analysis

Biotin-labeled synthetic RNAs (TREM2-ex4-bio and TREM2-ex4-d2-bio; Japan Bio Services Co., Ltd., Saitama, Japan) were mixed with Dynabeads MyOne Streptavidin T1 (Invitrogen) to generate biotin-labeled RNA-conjugated magnetic beads. The biotin-labeled RNA-conjugated magnetic beads were prepared using an amount of biotin-labeled RNA sufficient to saturate the magnetic beads and used for RNA pull-down assays. Nuclear fractions of PMA-differentiated THP-1 cells were extracted using NE-PER (Thermo Fisher Scientific). The nuclear extracts were diluted with 20 mM HEPES, mixed with Dynabeads MyOne Streptavidin T1, and rotated at 4 °C for 30 min. A portion of the resulting supernatant was collected as the input fraction, and the remainder was mixed with the respective biotin-labeled RNA-conjugated magnetic beads and rotated at 4 °C for 3 h. The beads were washed three times with PBS containing 1% Triton X-100 and cOmplete (Roche, Basel, Switzerland). The beads were subsequently washed with PBS containing 0.01% Triton X-100 and transferred to Protein LoBind tubes (Eppendorf, Hamburg, Germany). A portion of the beads was used for Western blotting, while the remaining beads were suspended in 30 μL of digestion buffer (50 mM ammonium bicarbonate and 0.05% ProteaseMAX). The suspension was supplemented with 3 μL of 100 mM dithiothreitol and incubated at 56 °C for 30 min with shaking. After cooling on ice, 3 μL of 100 mM iodoacetamide was added, and the samples were incubated at room temperature for 30 min with shaking in the dark. On-bead digestion was then performed overnight at 37 °C with 75 ng of trypsin (Promega, Madison, WI, USA) and 75 ng of lysyl endopeptidase (FUJIFILM Wako Pure Chemical, Osaka, Japan) per sample. Following digestion, the beads were separated magnetically, and the peptide-containing supernatants were collected. The resulting peptides were desalted and concentrated using StageTips (SP301; Thermo Fisher Scientific). The peptides were analyzed using a quadrupole-Orbitrap mass spectrometer (Q Exactive; Thermo Fisher Scientific) coupled to a Vanquish Neo system (Thermo Fisher Scientific) equipped with a C18 analytical column (0.1 × 125 mm; Nikkyo Technos, Tokyo, Japan).

LC-MS/MS analysis and data processing were performed as described previously [38]. Briefly, chromatographic separation was performed using 0.1% formic acid in water as mobile phase A and 0.1% formic acid in acetonitrile as mobile phase B. Raw LC-MS/MS data were processed using Proteome Discoverer version 2.4 (Thermo Fisher Scientific), and peptide identification was performed using Sequest HT against the UniProt human protein database. Label-free quantification was performed using the Minora Feature Detector with normalization based on the total peptide amount. Each peptide sample was analyzed in three technical replicates by LC-MS/MS.

### 2.10. RNA Pull-Down Assay

RNA pull-down assays were performed with minor modifications to a previously described method [39]. For the pull-down, Streptavidin Mag Sepharose beads (Cytiva, Marlborough, MA, USA) were incubated with biotinylated RNA (TREM2-ex4-WT-bio-RNA, TREM2-ex4-d2-bio-RNA, TREM2-ex4-8nt-1-bio-RNA, or TREM2-ex4-8nt-2-bio-RNA) to prepare the biotinylated RNA-conjugated beads. To prepare cell lysates, HEK293 cells were cultured to confluence in 10-cm dishes. The cell lysates were divided into four equal aliquots. Each aliquot was incubated with one of the four biotinylated RNA-bound beads for the RNA pull-down assay.

### 2.11. SDS-PAGE and Western Blotting

SDS-PAGE and Western blotting were performed as previously described [38]. Western blot signals were detected by chemiluminescence using Immobilon Forte Western HRP Substrate (Merck, Darmstadt, Germany) and visualized using a Luminograph III imaging system. The antibodies used in this study are listed in Supplementary Table S3. Original uncropped blots are provided in Supplementary Figure S3.

### 2.12. RNA Immunoprecipitation (RIP)

RIP was performed according to previously described methods [39]. In this study, we used an fl-TREM2 cell line transfected with EGFP-fused CELF1 or CELF2. EGFP-fused proteins were immunoprecipitated using Dynabeads Protein G magnetic beads (Thermo Fisher Scientific) conjugated with a mouse anti-GFP antibody (66002-1-Ig, Proteintech, Rosemont, IL, USA). A rabbit anti-GFP antibody (598MS, MBL, Tokyo, Japan) was used for Western blotting to confirm successful immunoprecipitation without detecting IgG chains. Co-immunoprecipitated RNA was amplified by RT-PCR using the primers listed in Supplementary Table S1.

### 2.13. Statistical Reproducibility

Statistical analyses were performed using Microsoft Excel, EZR [40], and R (version 4.5.1) with RStudio (version 2025.09.2). Comparisons between two groups were analyzed using Welch’s *t*-test in Microsoft Excel. Comparisons among three or more groups were evaluated using one-way analysis of variance (ANOVA) followed by Dunnett’s multiple comparison test in EZR. Normality was assessed using the Shapiro–Wilk test. For RT-PCR exon-skipping datasets that did not satisfy the assumption of normality, log transformation was applied prior to statistical analysis. If normality was not achieved after log transformation, non-parametric analysis was performed using the Kruskal–Wallis test followed by the Steel–Dwass multiple comparison test. For multiple-group comparisons, qPCR data were log-transformed and analyzed using one-way ANOVA followed by Dunnett’s test. For two-group comparisons, qPCR data were analyzed using Welch’s *t*-test. Data are expressed as mean ± standard deviation (SD), with error bars representing SD. The *n* value represents the number of independent experiments performed on different days. The detailed sample sizes and statistical tests are described in the figure legends. Graphs in Supplementary Figure S1 were generated using R in RStudio. Other graphs were generated using Microsoft Excel, with individual data points displayed.

## 3. Results

### 3.1. Identification of the Regulatory Region for TREM2 ex 4 Alternative Splicing

To identify the region responsible for the regulation of *TREM2* ex 4 alternative splicing, we generated a *TREM2* ex3-5 minigene covering the genomic region from ex 3 to ex 5 (Figure 1A). 

The minigene was transfected into HEK293 and HMC3 cells, and splicing patterns were analyzed by RT-PCR (Figure 1B). In both cell types, bands corresponding to transcripts containing ex 4, transcripts retaining both ex 4 and intron 4, and transcripts lacking both ex 4 and intron 4 were detected, although their relative levels differed between the cell types, confirming that the minigene undergoes alternative splicing.

Next, we divided ex 4 into nine regions and designed a gRNA targeting each region (Figure 1C). If a gRNA-targeted region contains an element important for splicing regulation, dCasRx recruited by the gRNA is expected to interfere with its function, thereby altering the splicing pattern. HEK293-dCasRx cells were transfected with each gRNA together with the *TREM2* ex3-5 minigene. RT-PCR analysis revealed that gRNAs 4-1, 4-2, 4-3, 4-7, 4-8, and 4-9 significantly promoted ex 4 skipping (Figure 1D,E). Among these, gRNA 4-8 showed the highest level of ex 4 skipping and was therefore selected for further analysis. To further narrow down the region targeted by gRNA 4-8, additional gRNAs (4-8a, 4-8b, and 4-8c) were designed (Figure 1F). These gRNAs were transfected into HEK293-dCasRx cells together with the *TREM2* ex3-5 minigene, and splicing assays were performed as described above. Among the three gRNAs, 4-8b most strongly promoted ex 4 skipping (Figure 1G). These results suggest that the 4-8b region of ex 4 contains a regulatory region involved in *TREM2* ex 4 alternative splicing.

### 3.2. Investigation of the Regulatory Region for ex 4 Alternative Splicing Using Deletion Mutants

Because each gRNA targets a region of approximately 20 nucleotides, multiple cis- regulatory elements may be present within each targeted region. To further narrow down the regulatory region, we generated several deletion mutant minigenes (Figure 2A). Based on the results described above, we focused on the ex4-8b region, which showed the strongest effect on ex 4 skipping, and generated three minigenes carrying distinct deletions within ex4-8b (*TREM2* ex3-5-d1, *TREM2* ex3-5-d2, and *TREM2* ex3-5-d3; Figure 2A). These minigenes were transfected into HEK293 cells, and splicing patterns were analyzed by RT-PCR. The d2 mutant significantly promoted ex 4 skipping (Figure 2B,C), whereas the d3 mutant significantly suppressed ex 4 skipping (Figure 2B,C). Hereafter, the regions deleted in the d2 and d3 mutants are referred to as the D2 and D3 regions, respectively.

We further investigated the D2 and D3 regions that significantly altered the splicing pattern (Figure 2D). Analysis of the splicing patterns of these deletion mutants revealed that the d2-2 mutant significantly promoted ex 4 skipping, whereas the d2-1 mutant showed a trend toward increased ex 4 skipping (Figure 2E,F). Taken together, these results suggest that the D2 region contains a cis-regulatory element involved in *TREM2* ex 4 alternative splicing. We also examined deletion mutants of the D3 region. The d3-1 mutant showed a significant decrease in ex 4 skipping, although the effect was weaker than that observed in the parental d3 mutant (Figure 2E,F). Thus, we focused on the D2 region, as it appeared more likely to contain a cis-regulatory element.

Because the experiments described above used a reporter minigene containing only the genomic region from *TREM2* ex 3 to ex 5, we next examined whether similar results could be obtained using a full-length *TREM2* minigene (Figure 2G). HEK293 cells were transfected with either the fl-*TREM2* minigene (fl-WT) or fl-*TREM2* ex4-d2minigene (fl-d2). Because *TREM2* transcripts lacking ex 3 undergo NMD-mediated degradation [30], cells were treated with CHX for 6 h before harvesting. RT-PCR analysis revealed that deletion of the D2 region promoted ex4 skipping, as well as concurrent skipping of ex 3 and ex 4 (Figure 2H). Thus, the fl-*TREM2* minigene yielded results similar to those obtained with the *TREM2* ex3-5 minigene, supporting the presence of an important cis-regulatory element within the D2 region.

### 3.3. Identification of Factors Regulating ex 4 Splicing

To identify proteins that bind to the D2 region, RNA pull-down followed by LC-MS/MS analysis was performed. Biotin-labeled synthetic RNAs covering the 4-8b region (WT) and the same RNA lacking the D2 region (d2) were generated (Figure 3A). These RNAs were conjugated to avidin beads and incubated with THP-1 nuclear extracts for RNA pull-down. Proteins bound to the beads were analyzed by LC-MS/MS, and multiple RBPs were identified (Figure 3B). Among these proteins, candidates were selected based on an abundance value greater than 1,000,000 and a detection level in WT that was at least twofold higher than that in d2. We also considered biological information and prioritized RBPs with known roles in pre-mRNA splicing. Among the top candidates, CELF1, CELF2, and DDX41 were selected for further analysis [31,41].

To validate the binding of these proteins to the D2 region (CUGGACUG), RNA pull-down assays were performed using the synthetic RNAs, and the pulled-down proteins were analyzed by SDS-PAGE. CELF1 and CELF2 showed weaker binding to the d2 RNA than to the WT RNA, suggesting that CELF1 and CELF2 bind to the D2 region (CUGGACUG). In contrast, DDX41 showed no substantial difference in band intensity between the WT and d2 RNAs (Figure 3C). Therefore, DDX41 was considered to bind to a sequence common to the WT and d2 RNAs. To further investigate the binding region of DDX41, synthetic RNAs corresponding to the 5′ and 3′ regions of d2 were designed (Figure 3D,E). RNA pull-down assays were then performed using these synthetic RNAs. DDX41 bound to both 8nt-1 and 8nt-2. In contrast, CELF1 and CELF2 bound to 8nt-1 but not to 8nt-2, suggesting that CELF1 and CELF2 have multiple binding sites within the first 16 nucleotides of the WT sequence (Figure 3F).

### 3.4. Effects of CELF1 and CELF2 on TREM2 Splicing

We investigated the effects of the candidate proteins CELF1 and CELF2 identified by LC-MS/MS on *TREM2* ex 4 splicing. siRNAs were transfected into an Flp-In HEK293 cell line (fl-TREM2 cell line) that stably expresses the genomic region of *TREM2* from the start codon to the stop codon [30], followed by RT-PCR analysis. Endogenous *CELF1* and *CELF2* in the fl-TREM2 cell line were significantly suppressed by siCELF1 and siCELF2, respectively (Supplementary Figure S1A,B). When the region spanning ex 3 to ex 5 was amplified, CELF2 knockdown significantly decreased ex 4 skipping. Simultaneous knockdown of CELF1 and CELF2 also significantly decreased ex 4 skipping, whereas CELF1 knockdown alone had no clear effect (Figure 4A,B). When the region spanning ex 2 to ex 5 was amplified, ex 4 skipping showed a similar trend to that observed for the ex 3–ex 5 region. CELF2 knockdown, either alone or in combination with CELF1 knockdown, significantly decreased ex 4 skipping (Figure 4C,D). Interestingly, CELF1 knockdown increased simultaneous skipping of ex 3 and ex 4, although the difference did not reach statistical significance (Figure 4C,E).

We next examined the effects of CELF1 and CELF2 knockdown on endogenous *TREM2* splicing in the human microglial cell line HMC3. Endogenous *CELF1* and *CELF2* in HMC3 were significantly suppressed by siCELF1 and siCELF2, respectively (Supplementary Figure S1C,D). Compared with the fl-TREM2 cell line, HMC3 cells showed more pronounced ex 4 skipping and simultaneous ex 3 and ex 4 skipping upon CELF1 knockdown, whereas CELF2 knockdown alone or simultaneous knockdown of CELF1 and CELF2 reduced ex 4 skipping (Figure 4F,G).

Finally, we performed an RNA immunoprecipitation (RIP) assay to investigate whether CELF1 and CELF2 associate with *TREM*2 pre-mRNA in cells. Western blot analysis confirmed that CELF1 and CELF2 were immunoprecipitated using an anti-GFP antibody (Figure 4H). RT-PCR detection of the *TREM2* intron 3–ex 4 region, which encompasses the d2 region, demonstrated the intracellular association of CELF1 and CELF2 with *TREM2* pre-mRNA (Figure 4I). Taken together, these results indicate that CELF1 and CELF2 may differentially regulate ex 4 splicing.

### 3.5. Effect of DDX41 on TREM2 Splicing

Because DDX41 was suggested to have multiple binding sites within the ex 4 region (Figure 3C,F), we investigated its effect on *TREM2* splicing. siDDX41 was transfected into the fl-TREM2 cell line, and the effect of DDX41 knockdown on ex 4 splicing was examined. Endogenous *DDX41* in fl-TREM2 cell line was significantly suppressed by siDDX41 (Supplementary Figure S1E). Knockdown of DDX41 significantly promoted ex 4 skipping (Figure 5A–D). We next examined the effect of DDX41 overexpression on *TREM2* splicing. In contrast to DDX41 knockdown, DDX41 overexpression suppressed ex 4 skipping (Figure 5E–H). No effect on ex 3 splicing was observed (Figure 5G), suggesting that DDX41 is involved in the regulation of *TREM2* ex 4 splicing. These results suggest that DDX41 promotes ex 4 inclusion in a dose-dependent manner, although its overall effect is relatively modest.

## 4. Discussion

In this study, we aimed to elucidate the molecular mechanisms regulating alternative splicing of *TREM2* ex 4 by identifying cis-regulatory regions within *TREM2* ex 4 and RBPs that bind to these regions. In experiments using dCasRx and gRNAs, targeting several regions within ex 4 altered its splicing pattern. In this study, we focused on the 4-8 region, which showed the strongest promotion of ex 4 skipping. Further narrowing down this region suggested that the D2 region (CUGGACUG) functions as a regulatory element for *TREM2* ex 4 alternative splicing. In contrast, deletion of the D3 region (UGGCCAU) reduced ex 4 skipping (Figure 2A). These findings suggest that the 4-8 region contains multiple sequence elements with opposing effects on splicing. Moreover, although we focused primarily on the 4-8 region in this study, other regions, including 4-1 and 4-9, were also implicated in splicing regulation (Figure 1D,E). This observation suggests that *TREM2* ex 4 contains multiple cis-regulatory elements distributed throughout the exon that may collectively contribute to the complex regulation of its alternative splicing. Further investigation of candidate regions other than 4-8 will therefore be necessary to gain a more comprehensive understanding of the mechanisms regulating *TREM2* ex 4 alternative splicing.

Based on the LC-MS/MS and RNA pulldown analyses, CELF1, CELF2, and DDX41 were identified as candidate regulators of *TREM2* ex 4 alternative splicing. Our previous study demonstrated that CELF1 and CELF2 are involved in the regulation of ex 3 splicing [31]. The results of the present splicing assays suggest that CELF1 and CELF2 regulate not only ex 3 splicing but also ex 4 splicing. CELF1 knockdown promoted ex 4 skipping, either alone or together with ex 3 skipping, depending on the cell type. In contrast, knockdown of CELF2 suppressed ex 4 skipping, suggesting that CELF2 promotes ex 4 skipping. Furthermore, simultaneous knockdown of CELF1 and CELF2 suppressed ex 4 skipping, suggesting that CELF2 may exert a stronger influence on ex 4 splicing than CELF1. The effect of CELF2 on promoting ex 4 skipping was consistent between fl-TREM2 (HEK293) and HMC3 cells. Both CELF1 and CELF2 have been reported to recognize UG-rich sequences, such as UG repeats and UGUU motifs [42,43,44]. Thus, although these two proteins recognize similar RNA sequences, it is particularly interesting that they exerted opposing effects on *TREM2* ex 4 alternative splicing. Regarding the biotinylated RNAs used for RNA pulldown, the D2 region contains two UG motifs, which may contribute to the binding of CELF1 and CELF2. However, UG motifs are also present in the flanking regions (8nt-1 and 8nt-2), and it therefore remains unclear whether the effects of CELF1 and CELF2 can be explained solely by the presence of UG motifs. Although CELF1 and CELF2 act in the same direction in the regulation of ex 3 splicing, they appear to exert opposing effects on ex 4 splicing, suggesting that the splicing outcome may depend on which of these proteins binds to the target site. CELF1 and CELF2 may compete for binding to the D2 region, and the balance between their protein levels may influence the splicing outcome of ex 4 (Supplementary Figure S2). These opposing effects may arise from differences in cofactors interacting with CELF1 and CELF2 or from differences in their cooperative interactions with other RBPs bound near the D2 region. Interestingly, TDP-43, an RBP associated with AD pathology [45], preferentially binds UG-rich RNA sequences, similar to CELF1 and CELF2 [46]. Whether TDP-43 is involved in *TREM2* splicing or shares splicing targets with CELF1 and CELF2 remains to be investigated.

DDX41 was also identified as a potential regulator of ex 4 splicing in this study. DDX41 encodes a DEAD-box RNA helicase that is predominantly localized in the nucleus. DDX41 has been reported to have several functions, including the resolution of R-loops and the processing of small nucleolar RNAs and ribosomal RNAs [47,48,49,50]. Knockdown of DDX41 promoted ex 4 skipping, suggesting that DDX41 promotes ex 4 inclusion (Supplementary Figure S2). Consistent with this observation, overexpression of DDX41 suppressed ex 4 skipping, further supporting its role in promoting ex 4 inclusion. Although DDX41 has been reported to bind to 5′ splice sites, its specific RNA-binding motif remains unclear [41]. Based on our RNA pulldown results, we speculate that DDX41 may recognize the CCA motif shared by the 8nt-1 and 8nt-2 regions; however, this possibility requires experimental validation.

This study has several limitations. Some experiments with small sample sizes may have limited statistical power to detect differences. Although RNA pulldown assays demonstrated the association of candidate DDX41 with nucleotides flanking the D2 region in vitro, its direct interaction with *TREM2* pre-mRNA in cells has not yet been established. While we observed changes in splicing upon modulation of CELF1, CELF2, or DDX41 expression, these changes do not necessarily indicate corresponding alterations in sTREM2 levels. The precise mechanisms by which CELF1, CELF2, and DDX41 regulate ex 4 splicing require further investigation. In addition, the effects of their depletion on ex 4 splicing were relatively modest. Other RBPs may also be involved in the regulation of ex 4 splicing. Further studies are therefore needed to investigate the combinatorial effects of these factors and to determine whether additional regulators with stronger effects on *TREM2* ex 4 splicing are involved.

## 5. Conclusions

We narrowed down a candidate region involved in the regulation of *TREM2* ex 4 splicing and identified CELF1, CELF2, and DDX41 as potential regulators. We previously reported that CELF1 and CELF2 also regulate *TREM2* ex 3 splicing, indicating that these RBPs are involved in the complex regulation of *TREM2* alternative splicing. Further studies are needed to elucidate the precise mechanisms by which these factors regulate *TREM2* splicing and to identify additional regulators that may be involved.

## Figures and Tables

**Figure 1:**
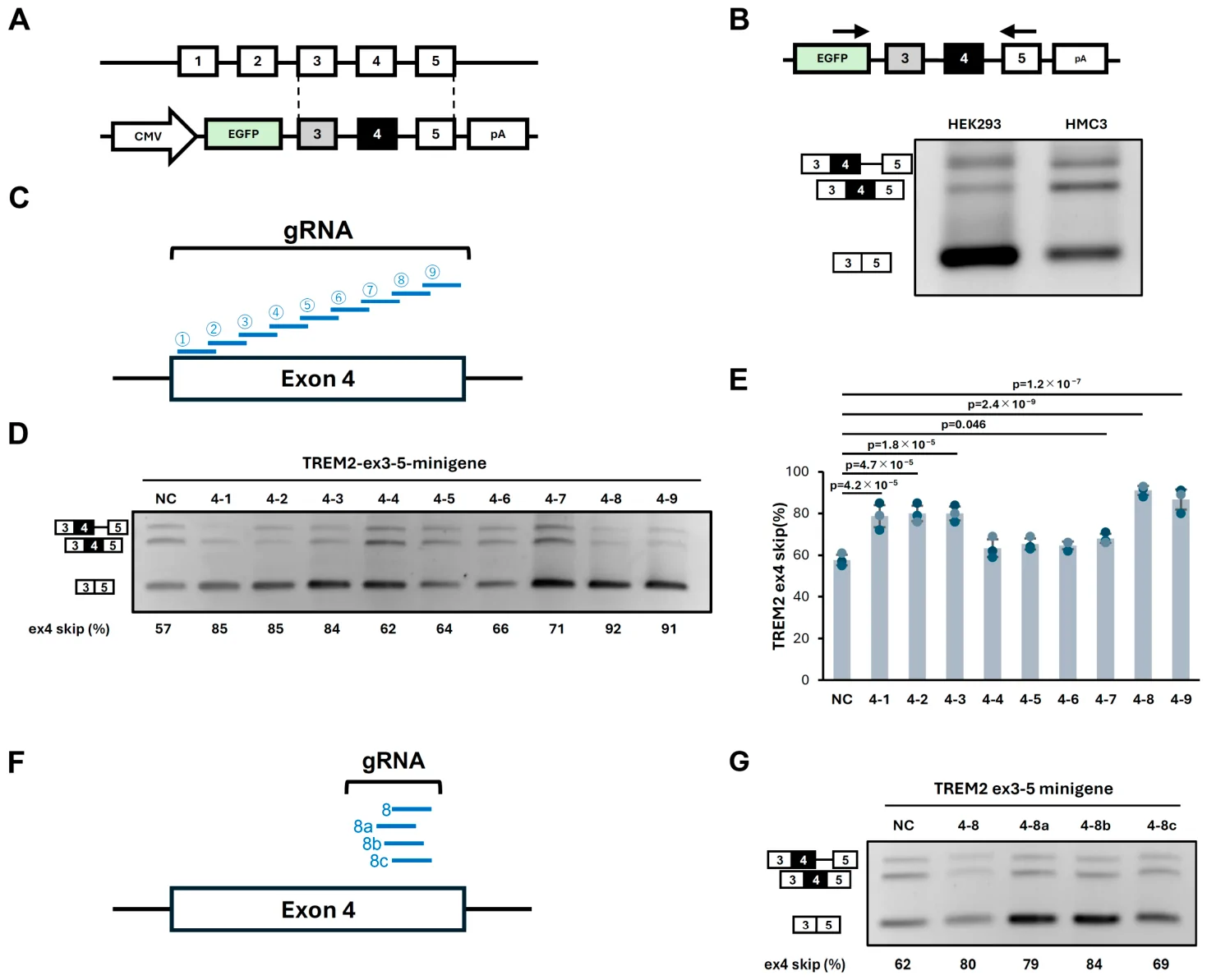
Identification of a candidate region involved in the regulation of *TREM2* ex 4 alternative splicing. (**A**) Schematic representation of the *TREM2* ex3-5 minigene reporter. (**B**) Representative agarose gel image showing the splicing patterns of the *TREM2* ex3-5 minigene in different cell lines (HEK293 and HMC3). Arrows indicate the positions of the primers used. (**C**) Schematic representation of the gRNAs (4-1–4-9) designed to target nine regions of ex 4. (**D**) Splicing assay using HEK293-dCasRx cells cotransfected with the *TREM2* ex3-5 minigene and the indicated gRNAs (4-1–4-9). Splicing products were detected by RT-PCR followed by agarose gel electrophoresis. The percentage of ex 4 skipping is shown below each lane. (**E**) Quantification of ex 4 skipping in (**D**). Statistical significance was assessed using Dunnett’s test. Error bars represent the S.D. (*n* = 3). (**F**) Schematic representation of the gRNAs targeting the 4-8 region. In addition to gRNA 4-8 shown in (**C**), gRNAs 4-8a, 4-8b, and 4-8c were designed to further narrow down the candidate region. (**G**) Splicing assay using HEK293-dCasRx cells cotransfected with the *TREM2* ex3-5 minigene and the indicated gRNAs (4-8, 4-8a, 4-8b, and 4-8c). Splicing products were detected by RT-PCR followed by agarose gel electrophoresis. The percentage of ex 4 skipping is shown below each lane.

**Figure 2:**
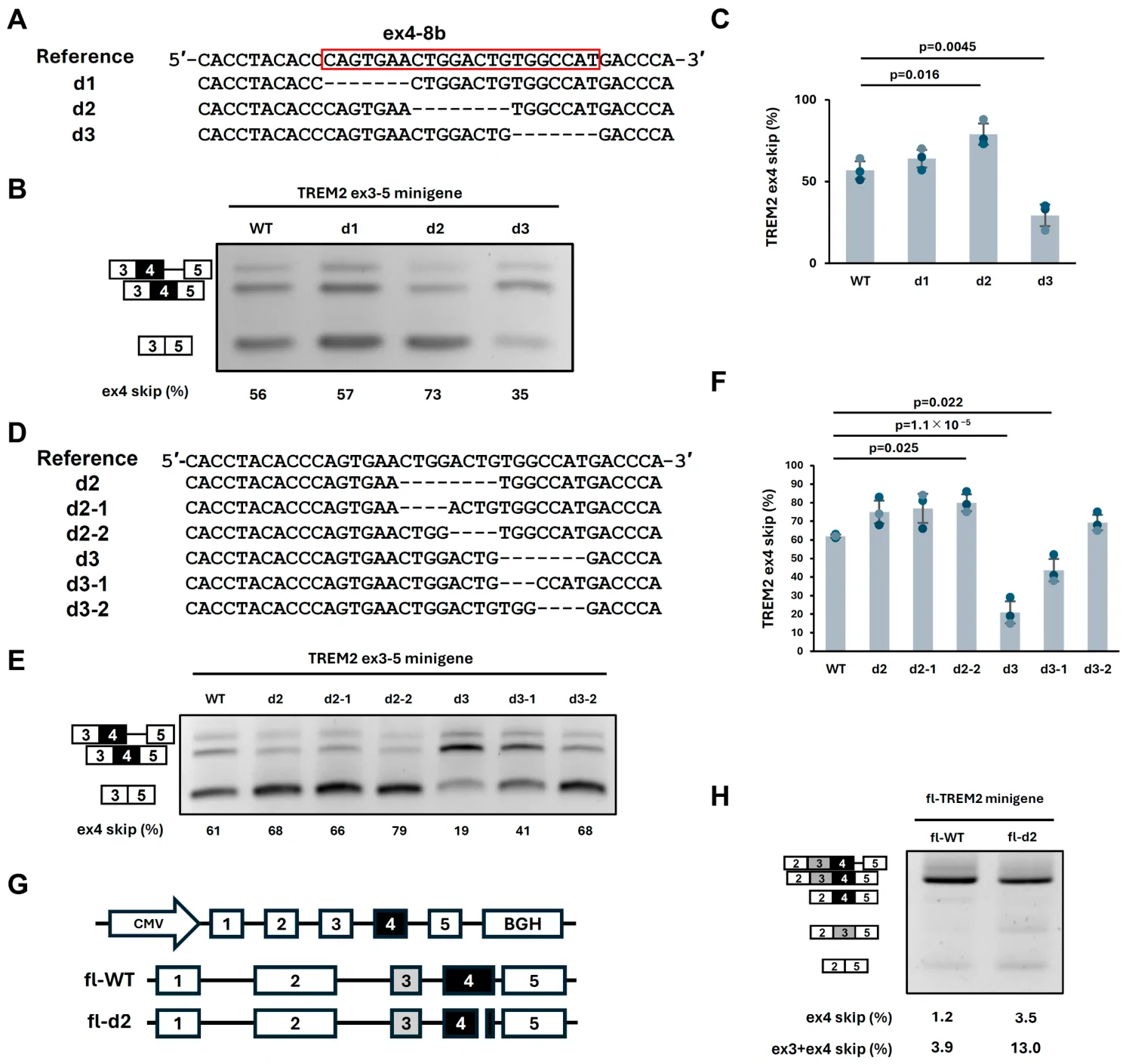
Fine mapping of the regulatory region involved in *TREM2* ex 4 alternative splicing. (**A**,**D**) *TREM2* sequences and the regions deleted in the indicated deletion mutants. (**B**,**E**) Splicing assays using HEK293 cells transfected with the indicated *TREM2* ex3-5 deletion mutants. Splicing products were detected by RT-PCR followed by agarose gel electrophoresis. The percentage of ex 4 skipping is shown below each lane. (**C**,**F**) Quantification of ex 4 skipping in (**B**) and (**E**), respectively. Statistical significance was assessed using Dunnett’s test. Error bars represent the S.D [(**C**), *n* = 3; (**F**), *n* = 3]. (**G**) Schematic representation of the fl-*TREM2* (fl-WT) and fl-*TREM2* ex4-d2 (fl-d2) minigene reporters. The region deleted in the fl-d2 minigene corresponds to that deleted in the d2 mutant shown in (**A**). (**H**) Splicing assay using HEK293 cells transfected with the fl-WT and fl- d2 minigenes. Splicing products were detected by RT-PCR followed by agarose gel electrophoresis. The percentages of ex 4 skipping alone and simultaneous ex 3 and ex 4 skipping are shown below each lane.

**Figure 3:**
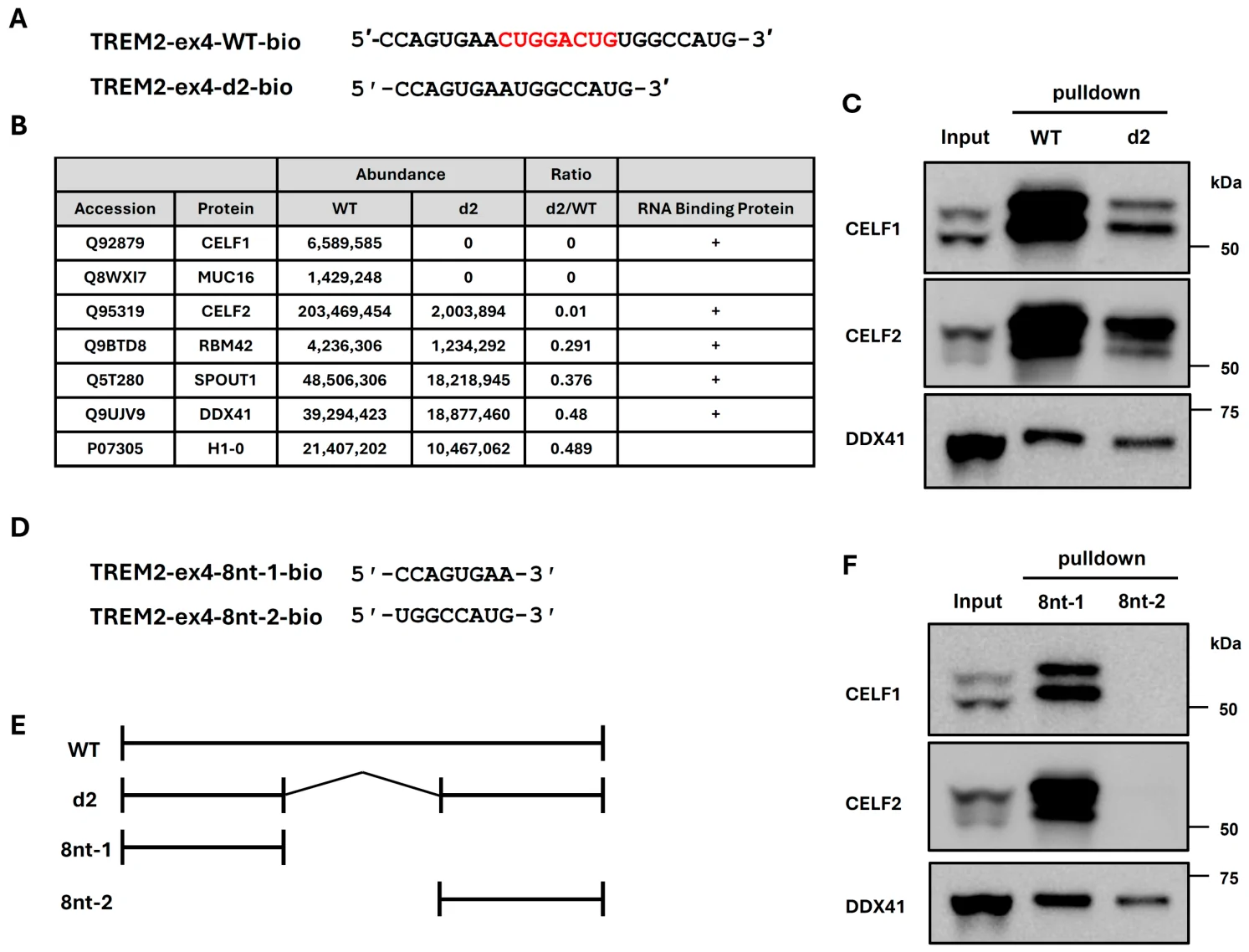
Identification of candidate regulators of *TREM2* ex 4 splicing. (**A**,**D**) Sequences of the synthetic RNAs used for RNA pulldown assays. The RNAs were biotinylated at their 3′ ends. The D2 sequence is shown in red. (**B**) Candidate RBPs binding to RNA containing the D2 region identified by LC-MS/MS analysis. Abundance values are expressed in arbitrary units. The complete dataset is provided as Supplementary Data. (**C**,**F**) Western blot analysis following RNA pulldown using HEK293 cell lysates. (**E**) Schematic representation of the synthetic RNAs used for the RNA pulldown assay.

**Figure 4:**
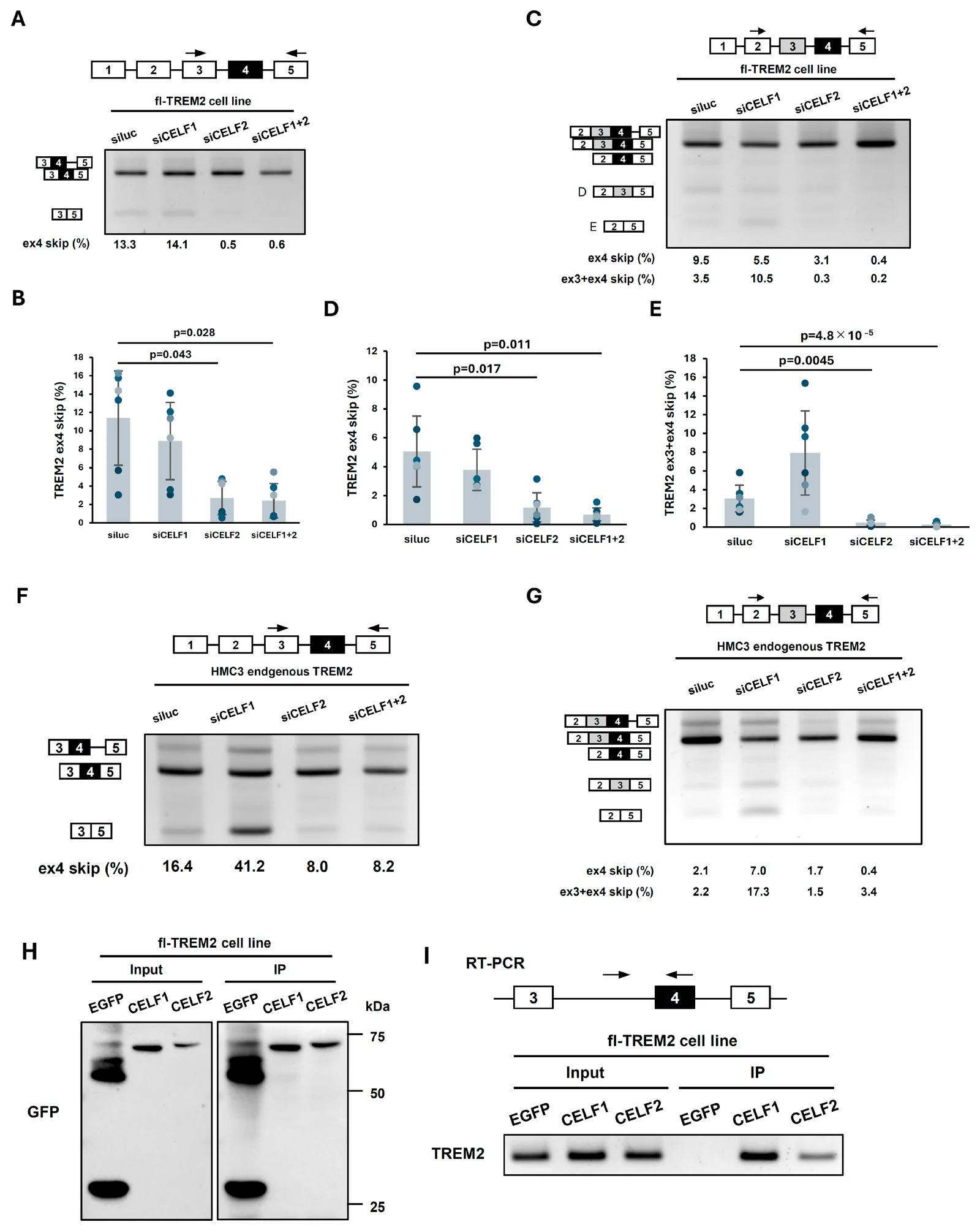
Effects of CELF1 and CELF2 on *TREM2* splicing. (**A**,**C**) The fl-TREM2 cell line was treated with the indicated siRNAs and subjected to splicing analysis. The regions spanning ex 3 to ex 5 (**A**) or ex 2 to ex 5 (**C**) were amplified by RT-PCR. (**B**,**D**,**E**) Quantification of the indicated splicing events. Error bars represent S.D, and *n* indicates the number of independent biological replicates. Statistical significance was assessed using the Kruskal–Wallis test followed by Steel’s multiple comparison test (**B**,**D**; *n* = 6) or one-way ANOVA followed by Dunnett’s test on log-transformed data (**E**; *n* = 6). (**F**,**G**) Splicing analysis of endogenous *TREM2* in HMC3 cells treated with the indicated siRNAs. The regions spanning ex 3 to ex 5 (**F**) or ex 2 to ex 5 (**G**) were amplified by RT-PCR. The percentages of ex 4 skipping and simultaneous ex 3 and ex 4 skipping are shown below each lane. (**H**) Western blot analysis confirms the immunoprecipitation of GFP-tagged CELF1 and CELF2 using an anti-GFP antibody. (**I**) RT-PCR analysis of cDNA synthesized from the immunoprecipitated RNA. Black arrows indicate the positions of the primers used to detect CELF1 and CELF2 binding. The knockdown efficiencies of the siRNAs in these samples are shown in Supplementary Figure S1. The primers and siRNAs used in this study are listed in Supplementary Tables S1 and S2, respectively. Arrows indicate the positions of the primers used.

**Figure 5:**
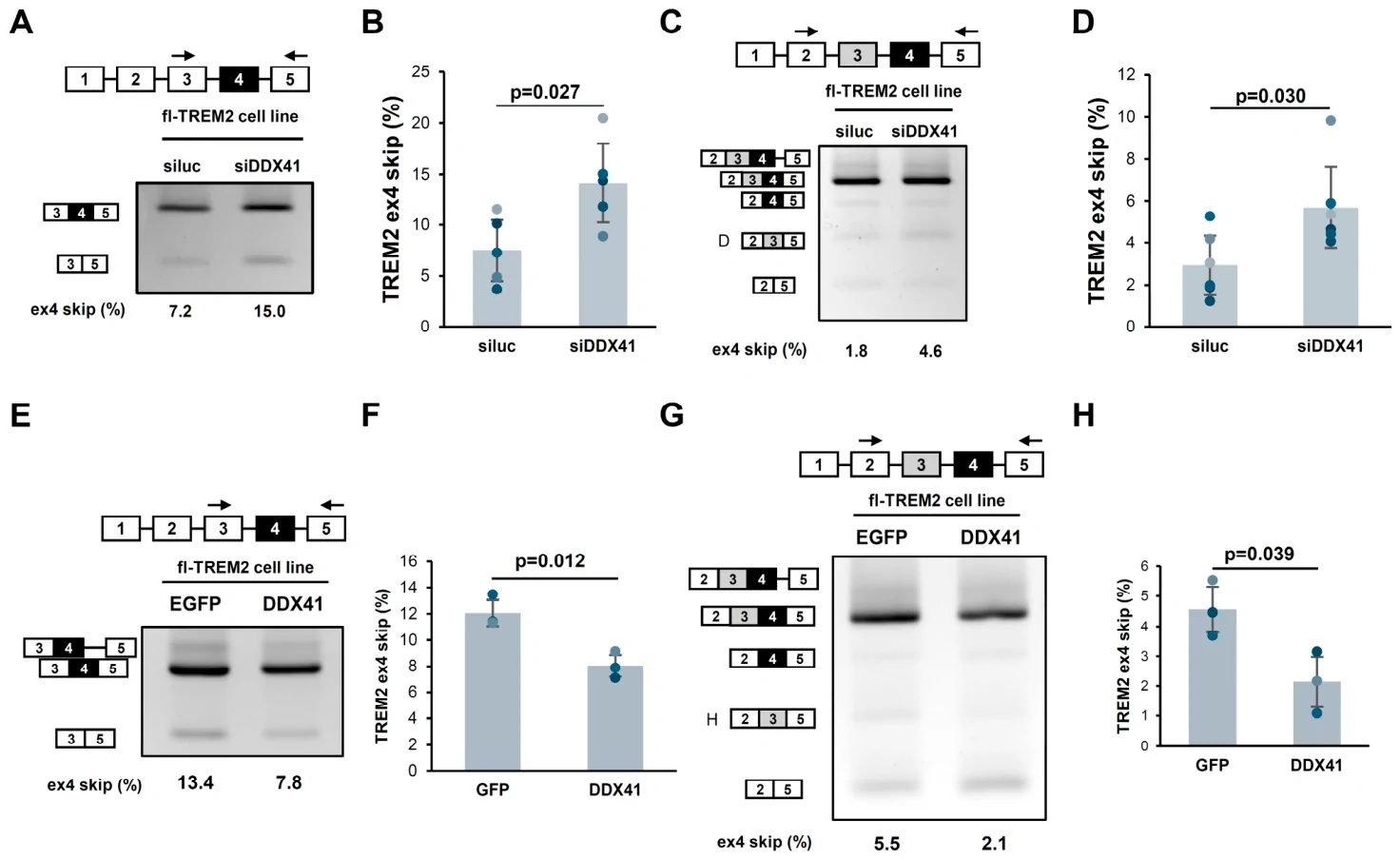
Effect of DDX41 on *TREM2* splicing. (**A**,**C**) The fl-TREM2 cell line was treated with the indicated siRNAs and subjected to splicing analysis. (**B**,**D**) Quantification of ex 4 skipping in (**A**) and (**C**), respectively. Error bars represent SD, and *n* indicates the number of independent biological replicates. Statistical significance was assessed using Welch’s *t*-test [(**B**), *n* = 5; (**D**), *n* = 6]. (**E**,**G**) Splicing analysis of the fl-TREM2 cell line transfected with EGFP-DDX41. Splicing products were detected by RT-PCR followed by agarose gel electrophoresis. (**F**,**H**) Quantification of ex 4 skipping in (**E**) and (**G**), respectively. Error bars represent SD, and *n* indicates the number of independent biological replicates. Statistical significance was assessed using Welch’s *t*-test [(**F**), *n* = 3; (**H**), *n* = 3]. The percentages of ex 4 skipping and simultaneous ex 3 and ex 4 skipping are shown below each lane. The knockdown efficiencies of the siRNAs in these samples are shown in Supplementary Figure S1. The primers and siRNAs used in this study are listed in Supplementary Tables S1 and S2, respectively. Arrows indicate the positions of the primers used.

## Data Availability

Data is contained within the article or Supplementary Materials.

## Supplementary Materials

The following supporting information can be downloaded at: https://www.mdpi.com/article/10.3390/genes17101188/s1, Supplementary Table S1: Primers used in this study; Supplementary Table S2: siRNAs and biotinylated RNAs used in this study; Supplementary Table S3: Antibodies used in this study; Supplementary Figure S1: Knockdown efficiencies of siRNAs used in this study; Supplementary Figure S2: Working model of *TREM2* exon 4 splicing regulation; Supplementary Figure S3: Uncropped original images. File S1. Data of Figure 1, Figure 2, Figure 3, Figure 4, Figure 5 and Figure S1.

## Abbreviations

The following abbreviations are used in this manuscript:

Aβ	amyloid-β
AD	Alzheimer’s disease
ADAM10/17	a disintegrin and metalloproteinase 10/17
APOE	apolipoprotein E
CELF	CUGBP Elav-like family
CHX	cycloheximide
CSF	cerebrospinal fluid
DDX41	DEAD-box helicase 41
EGFP	enhanced green fluorescent protein
gRNA	guide RNA
HEK293	human embryonic kidney cells 293
HMC3	human microglial clone 3
IgV	immunoglobulin variable
LC-MS/MS	liquid chromatography–tandem mass spectrometry
NMD	nonsense-mediated mRNA decay
PCR	polymerase chain reaction
RBP	RNA-binding protein
RT	reverse transcription
siRNA	small interfering RNA
sTREM2	soluble TREM2
TREM2	triggering receptor expressed on myeloid cells 2
TYROBP	TYRO protein tyrosine kinase binding protein
