# Supplementary material for: Identification of RNA-Binding Proteins Regulating the Alternative Splicing of TREM2 Exon 4
Source: Genes (Basel). 2026 Sep 25;17(10):1188. doi: 10.3390/genes17101188 (PMC13704697; doi:10.3390/genes17101188)
Supplement: Supplementary file 1 [file genes-17-01188-s001.zip › Yamada et al_Supplementary Materials_260918.pdf]

Primers for constuction

Constructs	primer	sequence (5' to 3')
gRNA 4-1	Fw	AAACGGATTTCTCCTTCCAAGAGGCTC
	Rv	AAAAGAGCCTCTTGGAAGGAGAAATCC
gRNA 4-2	Fw	AAACATGGAAGTGGGTGGGAAGGGGAT
	Rv	AAAAATCCCCTTCCCACCCACTTCCAT
gRNA 4-3	Fw	AAACGATGCAGGCCAGGAGGAGAAGGA
	Rv	AAAATCCTTCTCCTCCTGGCCTGCATC
gRNA 4-4	Fw	AAACCTGCTAGAATCTTGATGAGAAAG
	Rv	AAAACTTTCTCATCAAGATTCTAGCAG
gRNA 4-5	Fw	AAACGCTGCAGCCCAGAGGGCGCTGGC
	Rv	AAAAGCCAGCGCCCTCTGGGCTGCAGC
gRNA 4-6	Fw	AAACCCCTGGCTTCTGTCCATGCCAGG
	Rv	AAAACCTGGCATGGACAGAAGCCAGGG
gRNA 4-7	Fw	AAACCCAGTTCACTGGGTGGATGTGTC
	Rv	AAAAGACACATCCACCCAGTGAACTGG
gRNA 4-8	Fw	AAACTACCCTGGGTCATGGCCACAGTC
	Rv	AAAAGACTGTGGCCATGACCCAGGGTA
gRNA 4-9	Fw	AAACCTGGCAGAGTTTGGAGCTGATAC
	Rv	AAAAGTATCAGCTCCAAACTCTGCCAG
gRNA 4-8a	Fw	AAACACAGTCCAGTTCACTGGGTGGAT
	Rv	AAAAATCCACCCAGTGAACTGGACTGT
gRNA 4-8b	Fw	AAACATGGCCACAGTCCAGTTCACTGG
	Rv	AAAACCAGTGAACTGGACTGTGGCCAT
gRNA 4-8c	Fw	AAACTGGGTCATGGCCACAGTCCAGTT
	Rv	AAAAAACTGGACTGTGGCCATGACCCA
TREM2-ex3-5 minigene	BamHI-TREM2-ex3-Fw	AAAAAGGATCCCCCCTGGATCACCGGGATGCTG
	SalI-TREM2-ex5-Rv	AAAAAGTCGACTCAACCAGTCCCTGCTTCCAGGGT
TREM2-ex3-5-d1 minigene	TREM2-ex4-d1-Fw	GGACACATCCACCTGGACTGTGGCCATGACCCAGG
	TREM2-ex4-d1-Rv	GCCACAGTCCAGGTGGATGTGTCCCTGGCTTCTGT
	TREM2-int3-Fw2	GGAAGACTTCCATAATGCTTTGTAAAGAGC
	SalI-TREM2-ex5-Rv	AAAAGTCGACTCAACCAGTCCCTGCTTCCAGGGTC
TREM2-ex3-5-d2 minigene	TREM2-ex4-d2-Fw	CCACCCAGTGAATGGCCATGACCCAGGGTATCAGC
	TREM2-ex4-d2-Rv	GGGTCATGGCCATTCACTGGGTGGATGTGTCCCTG
TREM2-ex3-5-d2-1 minigene	TREM2-ex4-d2-1-Fw	AGTGAAACTGTGGCCATGACCCAGGGTATCAGCTC
	TREM2-ex4-d2-1-Rv	GGCCACAGTTTCACTGGGTGGATGTGTCCCTGGCT
TREM2-ex3-5-d2-2 minigene	TREM2-ex4-d2-2-Rv	GGCCACCAGTTCACTGGGTGGATGTGTCCCTGGCT
	TREM2-ex4-d2-2-Fw	AGTGAACTGGTGGCCATGACCCAGGGTATCAGCTC
TREM2-ex3-5-d3 minigene	TREM2-ex4-d3-Fw	TGAACTGGACTGGACCCAGGGTATCAGCTCCAAAC
	TREM2-ex4-d3-Rv	ATACCCTGGGTCCAGTCCAGTTCACTGGGTGGATG
TREM2-ex3-5-d3-1 minigene	TREM2-ex4-d3-1-Fw	ACTGGACTGCCATGACCCAGGGTATCAGCTCCAAA
	TREM2-ex4-d3-1-Rv	TCATGGCAGTCCAGTTCACTGGGTGGATGTGTCCC
TREM2-ex3-5-d3-2 minigene	TREM2-ex4-d3-2-Fw	ACTGTGGGACCCAGGGTATCAGCTCCAAACTCTGC
	TREM2-ex4-d3-2-Rv	CCTGGGTCCCACAGTCCAGTTCACTGGGTGGATGT
fl-TREM2-ex4-d2 minigene	TREM2-ex3-Fw	ACCCCCTGGATCACCGGGATGCTGGAGATCTCT
	TREM2-ex4-d2-Rv	GGGTCATGGCCATTCACTGGGTGGATGTGTCCCTG
	TREM2-ex4-d2-Fw	CCACCCAGTGAATGGCCATGACCCAGGGTATCAGC
	TREM2-ex3-int3-Fw	GCACAGCATCTCCAGGTACAGCGATGGGTCTTTCC
	NotI-TREM2-ex5-Rv	AAAAGCGGCCGCTCAACCAGTCCCTGCTTCCAGGG
EGFP-DDX41	BglII-DDX41-Fw	AAAAAGATCTATGGAGGAGTCGGAACCCGAACGGA
	SalI-DDX41-Rv	AAAAGTCGACTCAGAAGTCCATGGAGCTGTGGGCC

Primers for splicing assay

target	primer	sequence (5' to 3')
TREM2-ex3-5 minigene<br>TREM2-ex3-5-d1 minigene<br>TREM2-ex3-5-d2 minigene<br>TREM2-ex3-5-d2-1 minigene<br>TREM2-ex3-5-d2-2 minigene<br>TREM2-ex3-5-d3 minigene<br>TREM2-ex3-5-d3-1 minigene<br>TREM2-ex3-5-d3-2 minigene<br>fl-TREM2 minigene<br>fl-TREM2-ex4-d2 minigene<br>endogenous human TREM2	TREM2-ex3-Fw<br>TREM2-ex5-Rv<br>hTREM2-ex2-sp-Fw3<br>hTREM2-ex5-sp-Rv2	ACCCCCTGGATCACCGGGATGCTGGAGATCTCT<br>TCAACCAGTCCCTGCTTCCAGGGTC<br>GGTGGCACTCTCACCATTAC<br>TCACGTGTCTCTCAGCC

Primers for qPCR

target	primer	sequence (5' to 3')
ACTB	RT-ACTB-Fw2	CACCATTGGCAATGAGCGGTTC
	RT-ACTB-Rv2	AGGTCTTTGCGGATGTCCACGT
CELF1	RT-CELF1-Fw2	GCAGCAGATGCAGCAAATC
	RT-CELF1-Rv2	GCTGAAGGAGCTGCAAATAAAG
CELF2	RT-CELF2-Fw2	GAACCCTCCGCAGAGTAAAG
	RT-CELF2-Rv2	GGATGATGCATCCCAGGTAAA
DDX41	RT-DDX41-Fw	GCCAGAGGAGATTGAGAACTATG
	RT-DDX41-Rv	CACACGCTTTGTTGATGAAGG

Primers for RIP

target	primer	sequence (5' to 3')
TREM2	TREM2-int3-Fw	ATTCTAATCTGTAGGCCATCTCAAG
	hTREM2-ex4-sp-Rv2	CTGATACCCTGGGTCATGGCCACAGTCCAG

Supplementary Table S1. Primers used in this study.

siRNA

	**sense strand [19 mer(RNA) + TT]**
siCELF1-1413	GCUCAAACGUUCGAAGAAU+TT
siCELF2-1230	CCACCUUCCACAGGAAUUU+TT
siDDX41-658	GACAUGAUAGGCAUCGCUU+TT

biotinylated RNA

	**sequence (5' to 3')**
TREM2-ex4-bio	CCAGUGAACUGGACUGUGGCCAUG
TREM2-ex4-d2-bio	CCAGUGAAUGGCCAU
TREM2-ex4-8nt-1-bio	CCAGUGAA
TREM2-ex4-8nt-2-bio	UGGCCAUG

Supplementary Table S2. siRNAs and biotinylated RNAs used in this study.

primary antibodies	Source	Identifier	Dilution
Rabbit monoclonal DDX41	Cell Signaling Technology	#15076 (D3F1Z)	1:4000
Mouse monoclonal CUGBP1	santa cruz	sc-20003 (3B1)	1:2000
Rabbit monoclonal CELF2	abcam	ab186430 (EPR13374-2)	1:2000
Rabbit polyclonal GFP	MBL	598MS	1:3000
Mouse monoclonal GFP	proteintech	66002-1-Ig	-

secondary antibodies	Source	Identifier	Dilution
Goat anti-rabbit IgG (HRP)	Jackson ImmunoResearch	111-035-144	1:5000
Goat anti-mouse IgG (HRP)	Jackson ImmunoResearch	115-035-003	1:5000

Supplementary Table S3. Antibodies used in this study.

Supplementary Figure S1

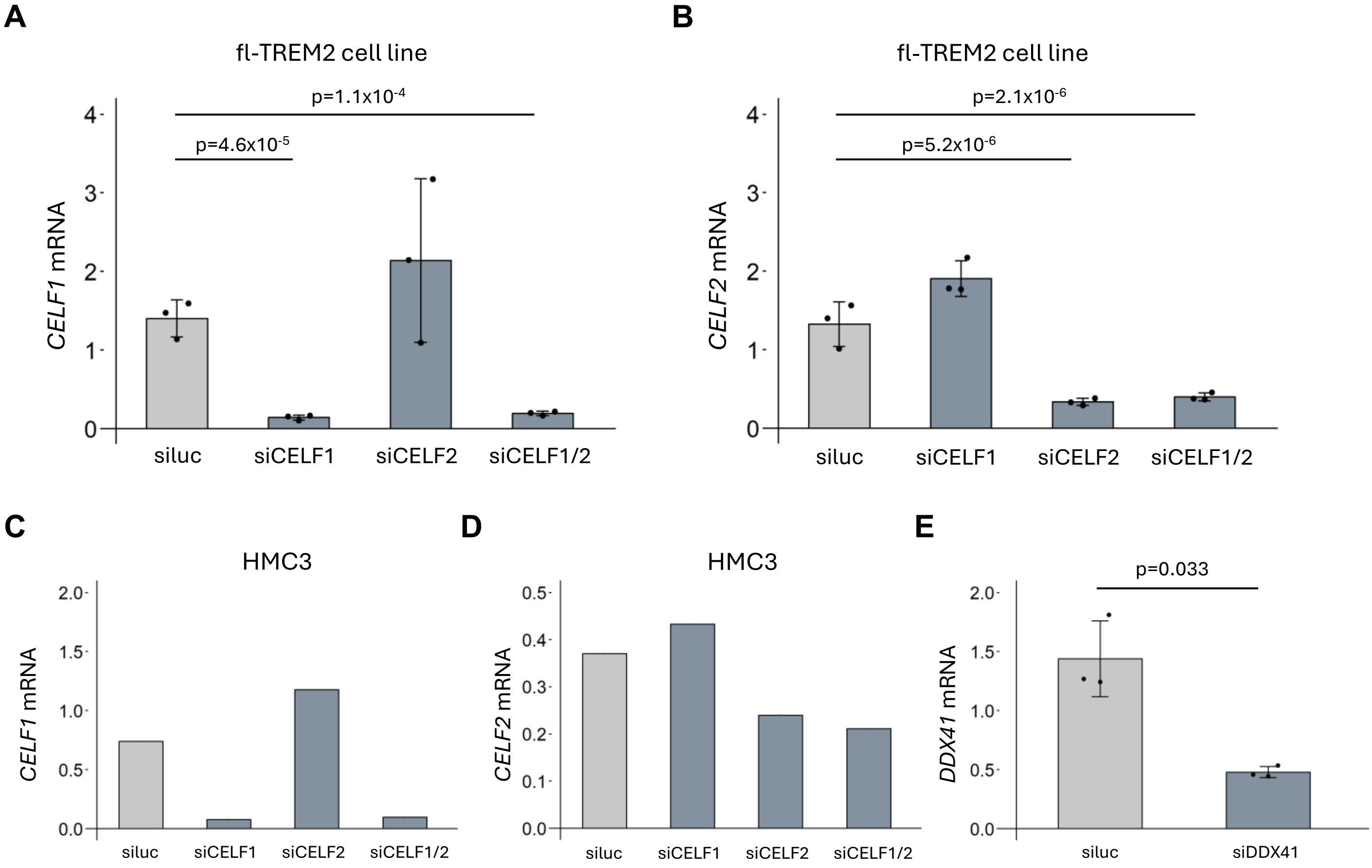

Supplementary Figure S1. Knockdown efficiencies of siRNAs used in this study.
(A, B) Bar graphs showing the knockdown efficiencies of siCELF1-1413 and siCELF2-1230 in fl-TREM2 cell line, corresponding to Figure 4A and C. p-values were determined using Dunnett's test after log transformation. Error bars represent S.D, n = 3. **(C, D)** Bar graphs show the knockdown efficiencies of siCELF1-1413, siCELF2-1230 in HMC3 cells, corresponding to Figure 4F and G. **(E)** The knockdown efficiency of siDDX41-658 in fl-TREM2 cell line. The expression levels of *CELF1*, *CELF2*, and *DDX41* were normalized to those of *ACTB*.
Error bars represent S.D, n = 3. Data were analyzed using Welch's t-test.

Supplementary Figure S2

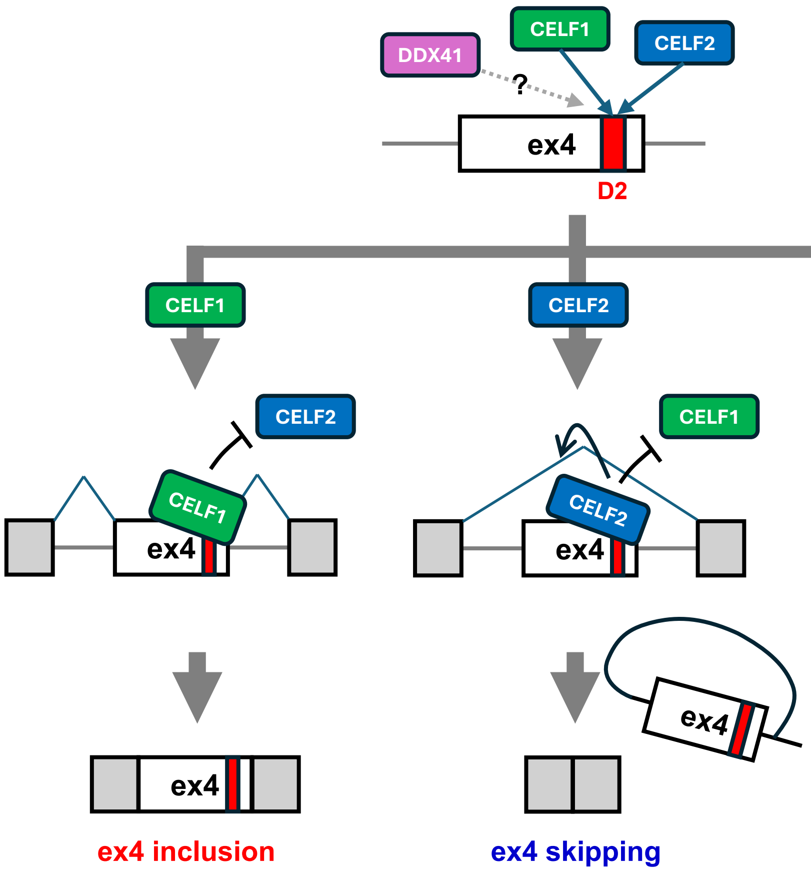

Supplementary Figure S2. Working model of *TREM2* exon 4 splicing regulation.
CELF1 and CELF2 both bind to the D2 region within *TREM2* exon 4. CELF1 promotes exon 4 inclusion, whereas CELF2 promotes exon 4 skipping, suggesting that they exert opposing effects on exon 4 splicing. The RNAi results suggest that CELF2 exerts a stronger influence on exon 4 splicing and that CELF1 may exert its effect by competing with CELF2. DDX41 appears to associate with regions flanking the D2 region rather than with the D2 region itself and promotes exon 4 inclusion in a dose-dependent manner. Further studies are needed to clarify the functional interactions among CELF1, CELF2, and DDX41.

Supplementary Figure S3

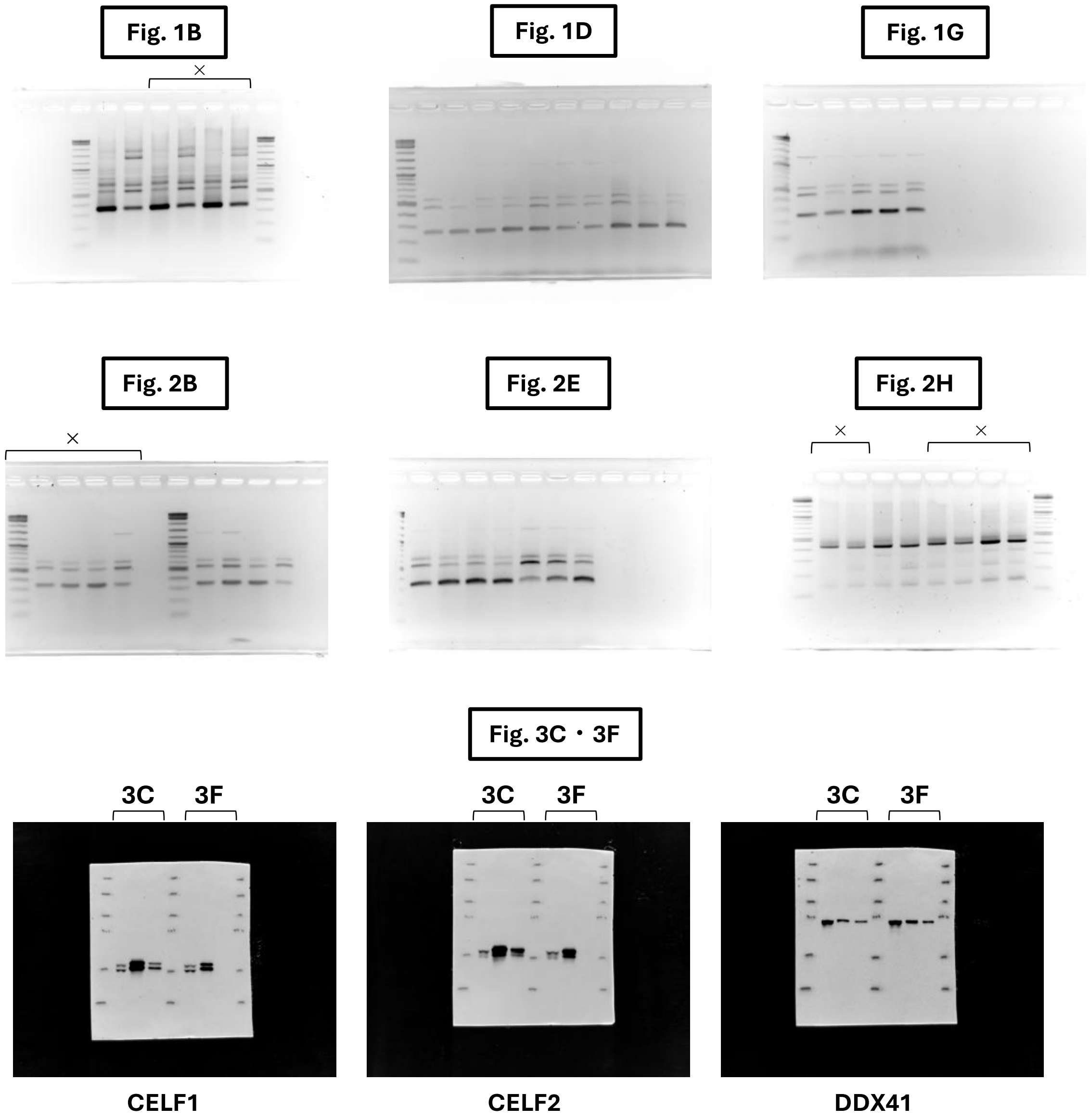

Supplementary Figure S3. Uncropped original images

Supplementary Figure S3 (continued)

Fig. 4A

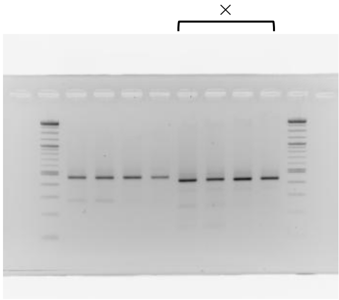

Fig. 4C

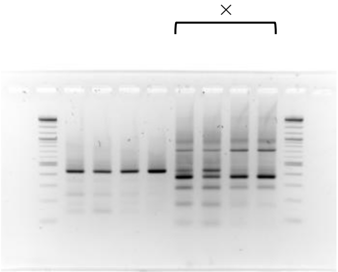

Fig. 4F

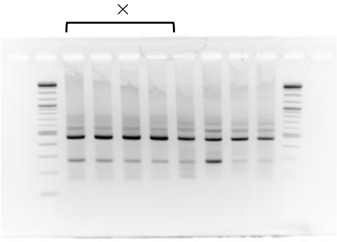

Fig. 4G

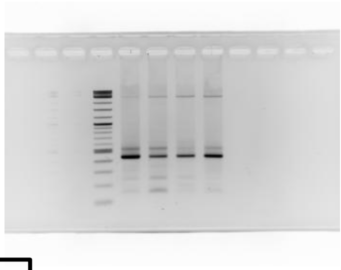

Fig. 4H

input

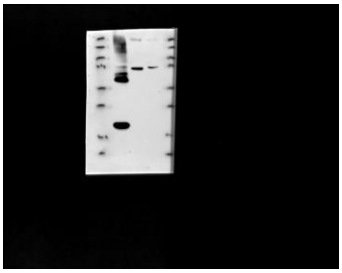

IP

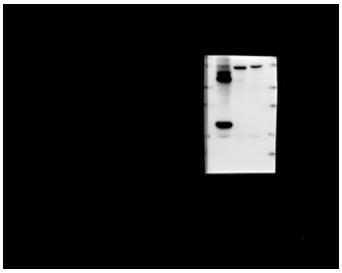

Fig. 4I

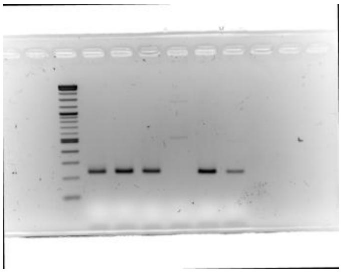

Fig. 5A

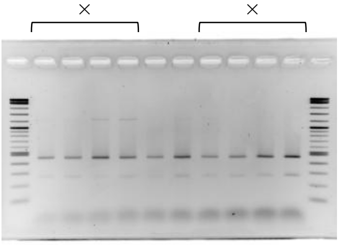

Fig. 5C

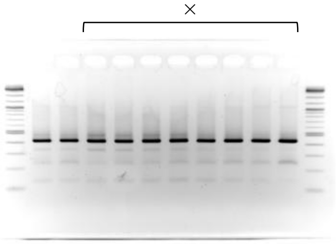

Fig. 5E

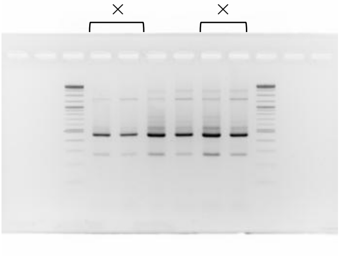

Fig. 5G

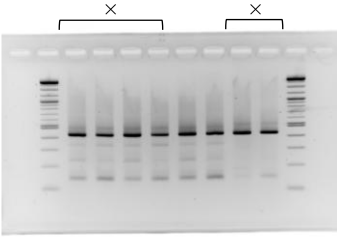

Supplementary Figure S3. Uncropped original images
